# Short-term changes in clinical parameters associated with telitacicept in pediatric IgA nephropathy: a single-center self-controlled observational study

**DOI:** 10.3389/fped.2026.1944384

**Published:** 2026-09-23

**Authors:** Xiaoxia Yu, Shijie Fu, Qiaohong Wang, Bing Chen, Xiaosong Shi, Shen Wu, Rirong Zhong, Jie Chen

**Affiliations:** 1 Department of Pediatrics, Shengli Clinical Medical College of Fujian Medical University, Fujian Provincial Hospital, Fuzhou University Affiliated Provincial Hospital, Fuzhou, Fujian, China; 2 Department of Pediatrics, Youxi General Hospital, Sanming, Fujian, China

**Keywords:** children, efficacy, IgA nephropathy, safety, telitacicept

## Abstract

**Background:**

Progressive IgA nephropathy (IgAN) in childhood presents challenges for treatment. Telitacicept has shown significant benefits in adult IgAN patients, warranting investigation into its potential efficacy in the pediatric population. This study was designed to evaluate the changes in clinical parameters before and after telitacicept treatment in children with IgA nephropathy (IgAN).

**Methods:**

A single-center, self-controlled before-and-after study was conducted. Children received subcutaneous injections of telitacicept once weekly, in addition to ongoing background therapy (including renin-angiotensin system inhibitors, sodium-glucose cotransporter 2 inhibitors, glucocorticoids, and/or other immunosuppressants). The primary clinical parameter was the change in 24-hour urinary protein (24-h UP) from baseline. Secondary clinical parameters included changes in serum albumin (ALB), immunoglobulins, CD19+ B-cell count, estimated glomerular filtration rate (eGFR), and immunosuppressant dosage. Adverse events were recorded.

**Results:**

Eleven children were included. The 24-h UP level began a non-significant decline at week 4 (*P* > 0.05) but continued to significantly decrease at weeks 12 and 24 (*P* < 0.05), whereas serum ALB levels tended to increase concurrently. Serum levels of IgG, IgA, and IgM exhibited a decreasing trend over time (*P* < 0.001). Compared with baseline, the reductions in all three immunoglobulin subtypes reached statistical significance at week 24 (*P* < 0.01), with IgA and IgM showing more pronounced decreases. The CD19+ B-cell count was significantly reduced at week 24 (*P* = 0.001). The eGFR remained stable throughout the treatment period (*P* > 0.05). The glucocorticoid dosage tended to decrease during follow-up (*P* < 0.05). Adverse events were mild, primarily upper respiratory tract infections (4 episodes) and local injection site reactions (1 episode), with no serious adverse events reported.

**Conclusion:**

In children with IgAN, telitacicept was associated with reduced proteinuria, facilitated immunosuppressant tapering, and demonstrated a favorable safety profile, while preserving renal function.

## 1 Introduction

Immunoglobulin A nephropathy (IgAN) is a glomerular disease characterized predominantly by IgA deposition and represents one of the most common primary glomerular diseases in children. It can occur at any age during childhood, but is more frequently observed among school-aged children and adolescents. Pediatric IgAN primarily manifests as various degrees of hematuria and/or proteinuria. Some children experience rapid disease progression and present with rapidly progressive glomerulonephritis or varying degrees of acute kidney injury (1). Currently, IgAN is generally considered to have a poor prognosis. A large cohort study involving 2,141 Chinese patients with IgAN reported a median renal survival time of 12.4 years, an annual incidence rate of end-stage kidney disease (ESKD) of 41.1 per 1,000 person-years, and a 15-year renal survival rate of only 40*%* (2). The pathogenesis of IgAN remains incompletely understood, with the “four-hit hypothesis” being the most widely accepted mechanism. The core steps involve the production of galactose-deficient IgA1 (Gd-IgA1), followed by the generation of anti-Gd-IgA1 antibodies and the subsequent formation of immune complexes that deposit in the glomerular mesangium. These deposits activate the complement system, promoting aberrant immune responses and leading to kidney injury (3). The production of Gd-IgA1 and the formation of corresponding immune complexes are key factors in the pathogenesis of IgAN. However, the mechanisms underlying Gd-IgA1 production have not been fully elucidated. McCarthy et al. reported that in BAFF-overexpressing transgenic mice, serum and mesenteric IgA levels were elevated, and IgA deposition was observed in the glomerular mesangium (4). Li W et al. reported that serum BAFF levels in patients with IgAN were positively correlated with IgA levels and the density of mesangial IgA deposition (5). Xin et al. demonstrated that serum BAFF levels were increased in the IgAN population and were associated with clinical severity and pathological features of the disease (6). Furthermore, APRIL gene expression is increased in the germinal centers of tonsils from IgAN patients and is correlated with serum Gd-IgA1 levels and disease severity (7). Therefore, targeting BAFF (BLyS)/APRIL represents a promising novel therapeutic strategy for IgAN.

Telitacicept is a novel, fully human TACI-Fc fusion protein generated by recombinant DNA technology that links the extracellular domain of the B-cell surface receptor TACI to the Fc domain of IgG. It binds to both BAFF and APRIL, effectively preventing their interaction with their cognate receptors (8). By neutralizing BAFF, telitacicept inhibits the continued development and maturation of immature B cells. Neutralization of APRIL suppresses the differentiation of mature B-cells into plasma cells and affects the release of autoantibodies from self-reactive plasma cells, thereby reducing the production of anti-Gd-IgA1 autoantibodies. Previous studies have reported that telitacicept has therapeutic efficacy in various immune-related kidney diseases, including lupus nephritis and adult IgAN (9). The present study aimed to investigate the short-term efficacy and safety of telitacicept in children with IgAN.

## 2 Methods

### 2.1 Study participants

A total of 11 children with IgAN who received telitacicept treatment at the Department of Pediatrics, Fuzhou University Affiliated Provincial Hospital, between March 2024 and October 2025, were enrolled. The inclusion criteria were as follows: (1) renal pathology consistent with the diagnostic criteria for IgAN (10); (2) age between 5 and 18 years; (3) estimated glomerular filtration rate (eGFR) ≥ 30 mL/(min·1.73 m^2^); (4) 24-hour urinary protein (24-h UP) ≥ 0.5 g/24 h after at least 3 months of treatment with renin-angiotensin-aldosterone system inhibitors (RAASis); (5) receipt of telitacicept treatment for at least 24 weeks; and (6) regular follow-up for more than 24 weeks. The exclusion criteria were as follows: (1) secondary IgAN, such as infection-associated nephritis, IgA vasculitis nephritis, or hepatitis B-associated nephritis; (2) other concurrent autoimmune or autoinflammatory diseases, such as systemic lupus erythematosus, rheumatoid arthritis, or inflammatory bowel disease; (3) concurrent primary or secondary immunodeficiencies, such as HIV infection, primary hypogammaglobulinemia, or persistent granulocytopenia; (4) concurrent chronic infectious diseases, such as chronic hepatitis B/C, chronic Epstein–Barr virus infection, chronic osteomyelitis, or Salmonella infection; (5) concurrent neoplastic diseases, such as leukemia, lymphoma, or solid tumors; and (6) allergy to telitacicept or any of its excipients.

### 2.2 Drug administration

Telitacicept was administered subcutaneously once weekly at a dosage of 80 mg per injection for patients weighing <40 kg and at a dosage of 160 mg per injection for those weighing ≥40 kg.

### 2.3 Data collection

(1) General information, including sex, age, height, weight, and blood pressure; (2) urinalysis, 24-h UP, serum creatinine (Scr), eGFR, IgG, IgA, IgM, and CD19+ B-cell count, and pathological classification at baseline and during follow-up; (3) use of specific medications, such as RAASi, sodium-glucose cotransporter 2 inhibitors (SGLT2i), glucocorticoids and other immunosuppressants; (4) dosage, frequency, and duration of telitacicept treatment; and (5) adverse reactions related to the therapeutic agent, such as injection site reactions and infections.

### 2.4 Clinical parameters

The primary clinical parameter was the change in 24-h UP from baseline to posttreatment. Secondary clinical parameters included changes in serum albumin (ALB), immunoglobulins (IgG, IgA, and IgM), CD19+ B-cell count, eGFR, immunosuppressant dosage from baseline to posttreatment,and adverse events.

### 2.5 Statistical analysis

Statistical analyses were performed using SPSS software (version 27.0). In this self-controlled before-and-after design study,categorical data are presented as numbers (*n*) and percentages (*%*). Continuous variables with a normal distribution are expressed as the mean ± standard deviation, while those with a nonnormal distribution are expressed as the median and interquartile range (IQR) (P25, P75). For comparisons between two groups of normally distributed continuous variables, the paired-sample t-test was used; for nonnormally distributed continuous variables, the paired-sample Wilcoxon signed-rank test was used. For comparisons among multiple groups, repeated-measures analysis of variance (ANOVA) was applied when homogeneity of variance was assumed, whereas the Friedman test was used when variances were unequal. A two-tailed *P*-value <0.05 was considered to indicate statistical significance.

### 2.6 Ethics statement

This study was approved by the Ethics Committee of the Provincial Hospital Affiliated with Fuzhou University (K2026-01-049). All procedures performed in this study involving human participants were in accordance with the ethical standards of the institutional research committee and with the 1964 Helsinki Declaration and its later amendments. The ethics committee approved the waiver of informed consent, as this was a retrospective study using deidentified patient data.

## 3 Results

### 3.1 Demographic characteristics and baseline data

A total of 11 children, including 8 males and 3 females, were included. The median (IQR) age was 12.0 (9.4, 13.0) years, and the median (IQR) disease duration was 3.5 (2.9, 4.4) years. The median (IQR) systolic blood pressure was 107 (103, 113) mmHg, and the median (IQR) diastolic blood pressure was 68 (65, 72) mmHg. Nine patients presented with hematuria and proteinuria, two with nephrotic syndrome, and two with intermittent gross hematuria. In terms of pathological findings, 11/11 patients had M1, 5/11 had E1, 2/11 had S1, 2/11 had T1-2, and 5/11 had C1-2. The median (IQR) 24-h UP levels at diagnosis, 1 month before baseline, and at baseline were 1.28 (1.01–2.05) g/d, 1.29 (1.03–1.36) g/d, and 1.59 (1.13–2.00) g/d, respectively. The 24-h UP level at baseline was significantly higher than that at 1 month before baseline (*W* = 7.5, *P* = 0.037). The mean baseline serum ALB concentration was 36.1 ± 4.5 g/L. All patients were classified as having stage 1 chronic kidney disease (CKD), with a mean baseline eGFR of 104.4 ± 10.8 mL/min/1.73 m^2^. The median (IQR) baseline values for IgG, IgA, and IgM were 7.33 (7.00, 8.44) g/L, 2.24 (1.63, 2.66) g/L, and 1.10 (0.74, 1.25) g/L, respectively. The median (IQR) baseline CD19+ B-cell count was 315 (281, 418) × 10⁶/L. The detailed baseline characteristics are presented in Tables 1, 2.

**Table 1:** Clinical characteristics and treatment parameters.

Patient no.	Age,years	Weight,kg	Clinical Kidney phenotype	Pathology, MEST-C	Baseline ALB, g/L	Baseline eGFR, mL/（min.1.73m^2^）	Baseline IgG, g/L	Baseline IgA, g/L	Baseline IgM, g/L	Baseline CD19+ B-cell,x10^6^/L
1	12.4	48	Hematuria and proteinuria	M1E0S0T0C1	38	97.8	7.23	1.37	1.27	245
2	8.3	33	Hematuria and proteinuria	M1E1S0T0C0	42	110.4	8.01	2.29	1.78	488
3	14.1	53	Nephrotic syndrome	M1E1S0T1C1	31	92.9	6.95	3.67	0.94	237
4	9.3	28	Hematuria and proteinuria	M1E0S0T0C1	35	130.3	7.74	1.89	1.35	404
5	7.8	27	Hematuria and proteinuria	M1E1S0T0C0	30	94.9	5.99	2.45	0.66	289
6	13.7	39	Nephrotic syndrome	M1E0S0T0C0	29	95.9	7.05	2.93	0.57	367
7	11.3	39	Hematuria and proteinuria	M1E1S0T0C0	41	109.5	9.22	1.28	1.1	432
8	9.6	35	Hematuria and proteinuria	M1E0S0T1C0	39	102.7	9.86	1.25	0.51	476
9	15.4	63	Hematuria and proteinuria	M1E0S1T0C1	37	98.8	7.33	2.87	0.82	273
10	11.3	38	Hematuria and proteinuria	M1E1S0T0C1	35	103.3	6.51	2.24	1.23	305
11	12.3	52	Hematuria and proteinuria	M1E0S1T0C0	40	112.5	8.87	1.96	1.14	315

Patient no.	24-h UP at diagnosis,g/24h	24-h UP at glucocorticoid initiation,g/24h	24-h UP at 1 month before baseline,g/24h	Baseline 24-h UP,g/24h	Glucocorticoid type and dosage at 1 month before baseline,mg/d	Glucocorticoid type and dosage at baseline,mg/d	Glucocorticoid type and dosage at week 24 of telitacicept treatment,mg/d	Cumulative glucocorticoid use duration before baseline, weeks	Concomitant medications at baseline	Concomitant medications at week 24 of telitacicept treatment	Telitacicept dosage, mg/dose
1	0.84	1.25	0.75	1.12	P，27.5	P，30.0	P，6.1	26	P + MMF + Benazepril	P + MMF + Benazepril	160
2	1.17	1.17	1.26	1.56	P，30.0	P，25.0	P，5.0	49	P + Benazepril	P + Benazepril	80
3	3.82	3.82	2.11	3.78	P，55.0	P，50.0	P，10.0	57	P + Tac + DAPA	P + DAPA	160
4	1.28	1.28	1.37	1.13	P，20.0	P，15.0	P，0	35	P + MMF + Benazepril	MMF + Benazepril	80
5	0.72	1.07	1.34	1.89	P，25.0	P，20.0	P，0	32	P + Valsartan	Valsartan	80
6	2.54	2.54	1.29	2.65	P，35.0	P，35.0	P，7.5	81	P + DAPA	P + DAPA	80
7	1.56	1.56	0.79	1.66	P，10.0	P，12.5	P，5.0	39	P + Benazepril	P + Benazepril	80
8	1.30	1.03	0.66	1.59	P，25.0	P，25.0	P，0	28	P + Benazepril	Benazepril	80
9	2.10	2.10	1.55	0.98	P，35.0	P，30.0	P，5.0	77	P + Benazepril	P + Benazepril	160
10	1.67	1.67	1.38	1.12	P，30.0	P，20.0	P，5.0	41	P + MMF + Valsartan	P + Valsartan	80
11	0.89	0.83	0.88	1.56	P，25.0	P，22.5	P，0	23	P + Tac + Benazepril	Tac + Benazepril	160

P, prednisone, MMF, mycophenolate mofetil, Tac, tacrolimus, DAPA, Dapagliflozin. *P = 0.037 vs. baseline (Wilcoxon signed-rank test: *W* = 7.5). #*P* = 0.078 vs. baseline (Wilcoxon signed-rank test: *W* = 7).

**Table 2:** Comparisons of laboratory parameters at weeks 4, 12, and 24 (*n* = 11).

Clinical parameters	Baseline	Week 4 of medication	Week 12 of medication	Week 24 of medication	Statistic	*P*
24-h UP (g/24 h)	1.59 (1.13, 2.00)	1.34 (1.03, 1.78)	0.45 (0.33, 0.83)**	0.43 (0.30, 0.66)**	24.49	<0.001
ALB(g/L)	36.1 ± 4.5	37.2 ± 3.2	40.8 ± 2.8*	41.1 ± 2.2*	3.909	<0.001
eGFR(mL/min/1.73m^2^)	104.4 ± 10.8	106.2 ± 6.5	101.3 ± 7.3	108.9 ± 9.8	1.835	0.183
IgG (g/L)	7.33 (7.00, 8.44)	-	6.14 (5.61, 7.28)*	5.33 (4.74, 6.04)**	20.18	<0.001
IgA (g/L)	2.24 (1.63, 2.66)	-	1.87 (1.50, 2.09)	1.57 (1.21, 1.75)**	14.73	<0.001
IgM (g/L)	1.10 (0.74, 1.25)	-	0.96 (0.65, 1.12)	0.63 (0.44, 0.85)**	16.91	<0.001
CD19+ B-cells (×10^6^/L)	315 (281, 418)	-	291 (241, 344)	227 (169, 260)**	12.18	0.001

Compared with baseline, *P < 0.05, **P < 0.01. Serum immunoglobulin levels (IgG, IgA, and IgM) were not assessed at week 4 because of incomplete data.

All patients received glucocorticoids and RAASi/SGLT-2i inhibitors prior to enrollment. Among them, 3 patients were concomitantly treated with mycophenolate mofetil (MMF), and 2 patients received tacrolimus (Tac). The median (IQR) glucocorticoid dose (prednisone-equivalent) was 27.5 (22.5–32.5) mg/d at 1 month before baseline and 25.0 (17.5–32.5) mg/d at baseline, with no statistically significant difference between the two time points (*W* = 7, *P* = 0.078) (Table 1). The formulations and dosages of MMF, Tac, RAASi, and SGLT-2i remained unchanged from 1 month before baseline to baseline. All patients received telitacicept treatment for at least 24 weeks. Four patients received a single dose of 160 mg, and seven patients received a single dose of 80 mg, with no dose adjustments during the treatment period. The longest interruption of telitacicept treatment was less than two weeks. The baseline treatment characteristics are presented in Table 1.

### 3.2 Primary clinical parameter

The 24-hour urinary protein (24-h UP) levels tended to decrease at each follow-up time point after telitacicept treatment, with statistically significant differences observed at weeks 12 and 24 (*P* < 0.01), as shown in Table 2. At the 24-week follow-up endpoint, the median 24-h UP was less than 0.5 g/24 h [0.43 (0.30, 0.66) g/24 h], representing a 73.0% reduction from baseline. Among the 11 patients, 2 (18.2%) achieved a 24-h UP concentration less than 0.15 g/24 h, and 5 (45.4%) had 24-h UP concentrations between 0.15 g/24 h and 0.5 g/24 h, as illustrated in Figure 1A.

**Figure 1:**
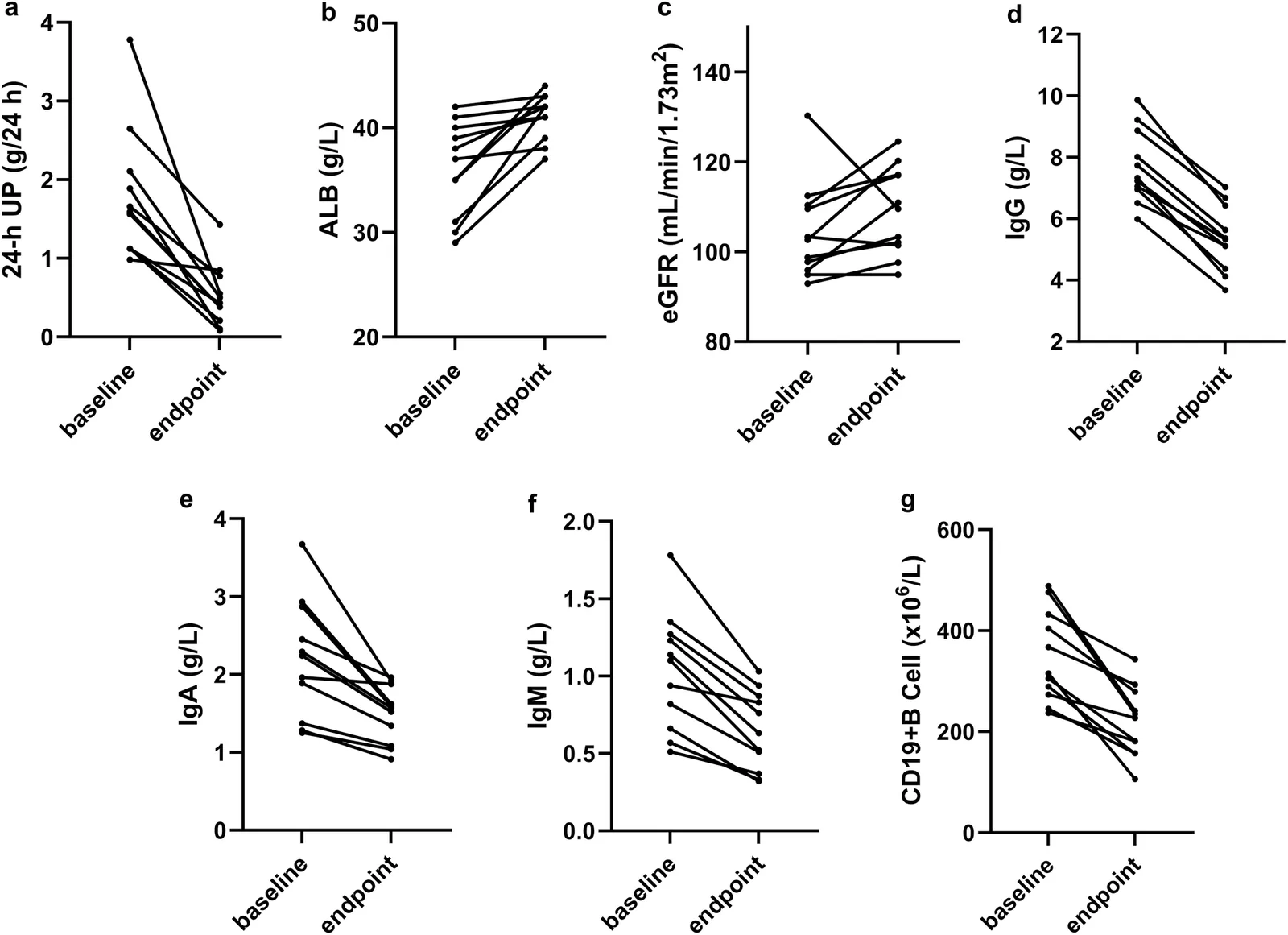
Trends in laboratory parameters. At the endpoint, **(a)** All children showed a reduction in 24-h UP compared to baseline. Among them, 2/11 cases achieved a 24-h UP below 0.15 g/24 h, and 5/11 cases had 24-h UP between 0.15 g/24 h and 0.5 g/24 h. **(b)** Serum ALB levels increased in all children compared to baseline. **(c)** All children had eGFR within the normal range. **(d)** Serum IgG levels decreased in all children, with 10/11 cases showing IgG below 7 g/L, and the lowest value reaching 3.68 g/L. **(e)** Serum IgA levels decreased in 10/11 children, but all remained above 0.7 g/L, with the lowest value at 0.91 g/L. **(f)** Serum IgM levels decreased in all children, with the lowest value reaching 0.32 g/L. **(g)** CD19+ B-cell counts decreased in all children, with the lowest value reaching 106×10⁶/L.

### 3.3 Secondary clinical parameters

Serum albumin (ALB) levels tended to increase at each follow-up time point after telitacicept treatment, with statistically significant differences observed at weeks 12 and 24 (*P* < 0.05), as shown in Table 2. Immunoglobulin levels (IgG, IgA, and IgM) tended to decrease at all follow-up time points, with significant decreases from baseline at week 24 (*P* < 0.05). At the 24-week follow-up endpoint, all 11 patients (100%) had decreased IgG levels, with 10 of 11 patients having IgG concentrations less than 7 g/L (lowest value: 3.68 g/L). IgA levels decreased in 10 of 11 patients (90.9%), with all values remaining above 0.7 g/L (lowest value: 0.91 g/L). All 11 patients (100%) had decreased IgM levels, with the lowest value reaching 0.32 g/L. The CD19+ B-cell count also tended to decrease at each follow-up time point, with a significant decrease observed at week 24 (*P* < 0.05). The estimated glomerular filtration rate (eGFR) did not significantly change at any time point (*P* > 0.05), and all patients maintained their eGFR values within the normal range (Figure 1).

### 3.4 Immunosuppressant use

The glucocorticoid dosage (prednisone or equivalent conversion) tended to decrease at all follow-up time points after telitacicept treatment [*χ*^2^(3) = 29.47, *P* < 0.001], with a mean reduction of 86.4*%* by the end of follow-up (Figure 2). At week 24, 4 of 11 patients (36.4*%*) discontinued glucocorticoids, while 1 of 11 patients (9.1*%*) experienced a temporary dose increase due to infection, followed by a dose reduction after disease remission. Additionally, 1 of 11 patients (9.1%) discontinued MMF, and 1 of 11 patients (9.1*%*) discontinued Tac.

**Figure 2:**
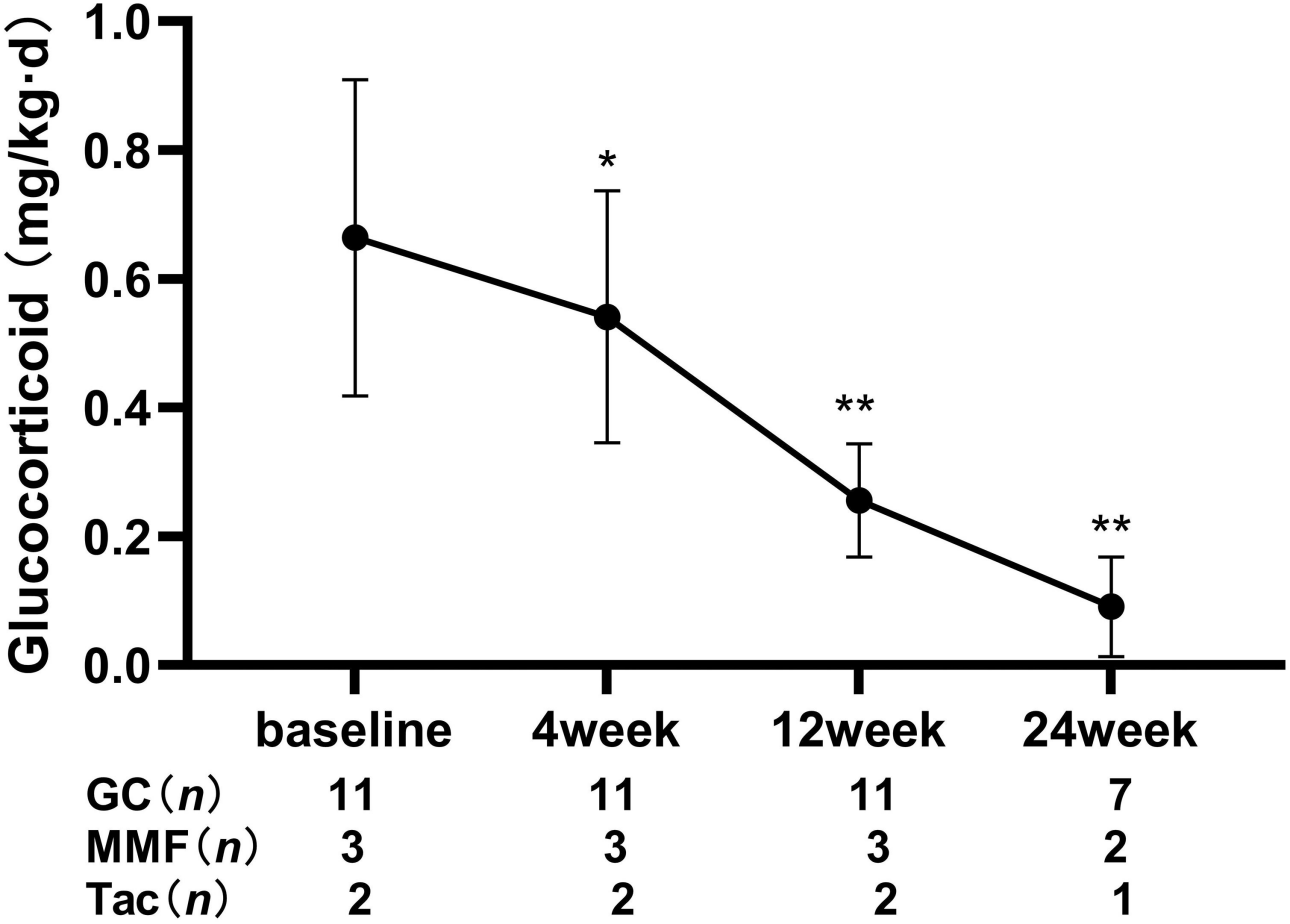
Changes in glucocorticoid dosage. The dosage of glucocorticoids showed a decreasing trend at each follow-up time point, with a reduction of 86.4% from the baseline value at the end of follow-up. At the endpoint, one patient discontinued MMF, and one patient discontinued Tac. GC, glucocorticoids; MMF, mycophenolate mofetil; Tac, tacrolimus. Compared with baseline, * *P* < 0.05, ** *P* < 0.01.

### 3.5 Safety analysis

No deaths occurred among the enrolled patients during the follow-up period. Upper respiratory tract infections occurred in 3 patients (4 episodes), and injection site reactions (local redness and swelling) occurred in 1 patient (1 episode). These adverse events did not lead to treatment interruption. No serious adverse events were reported.

## 4 Discussion

The treatment of children with IgAN is characterized by considerable heterogeneity. Multiple international guidelines recommend RAASis as the foundational therapeutic regimen and emphasize a tiered management approach based on the severity of proteinuria and pathological findings (11–13). In clinical practice, most pediatric experts tend to prefer glucocorticoids or other immunosuppressants for disease control. The IPNA guidelines for IgAN recommend that a 4 to 6month course of glucocorticoid therapy may be considered for children with IgAN at risk of clinical progression, and that combination with other immunosuppressants may be considered for those with rapidly progressive IgAN or those unresponsive to glucocorticoid monotherapy (12). The KDIGO 2025 guidelines for pediatric IgAN are consistent with the IPNA recommendations (13). Although the pediatric population appears to tolerate glucocorticoids far better than adults do, the risks and benefits of immunosuppressant use must still be carefully weighed in clinical practice. Early results from the TESTING study in adults demonstrated that full-dose glucocorticoid regimens carried a substantial risk of fatal infections; the first phase of the study was interrupted because of unacceptable rates of fatal infections, and the study endpoint was only reached after a reduced-dose regimen was adopted (14). An earlier study, the STOP study, failed to demonstrate better outcomes in IgAN patients receiving glucocorticoid therapy (15).

In recent years, B-cell-targeting biologic agents have shown potential benefits for the treatment of IgAN, although outcomes vary depending on the specific target. A small randomized controlled trial involving 34 adult patients compared rituximab with supportive therapy and reported no significant difference in proteinuria changes between the two groups after 12 months or any improvement in the eGFR (16). In contrast, the efficacy of telitacicept, which targets both BAFF and APRIL, has been demonstrated. A phase II clinical trial (NCT04291781;registered February 27, 2020, on ClinicalTrials.gov) conducted in Chinese adults with IgAN revealed that patients receiving 240 mg of telitacicept weekly achieved a 49% reduction in proteinuria at 24 weeks, with no significant decline in renal function during follow-up (17). In another multicenter, randomized, double-blind, placebo-controlled phase III trial (NCT05799287; registered March 5, 2023,on ClinicalTrials.gov) involving 318 adult patients with IgAN, treatment with telitacicept resulted in a 58.9% reduction from baseline in the 24-hour urine protein-to-creatinine ratio at week 39, markedly surpassing the 8.8% reduction observed in the placebo group. The percent change from baseline in estimated glomerular filtration rate was −1.0% (95% CI, −3.2 to 1.2) in the telitacicept group, compared with −7.7% (95% CI, −9.9 to −5.2) in the placebo group (18). High-quality clinical studies on pediatric IgAN are scarce. Only a few case reports and self-controlled before-and-after studies in Chinese populations have preliminarily suggested an association between telitacicept and reduced proteinuria (19–23). Jin Y et al. conducted a retrospective, observational, single-center study including 12 children with IgAN who received telitacicept, with a median follow-up of 9 months. The median (IQR) 24-h UP decreased significantly from 0.98 (0.5–9.8) g/1.73 m^2^ at baseline to 0.36 (0.08–2.49) g/1.73 m^2^ at 12 months (*P* < 0.01), with 7 patients (58.3%) achieving complete remission at the last follow-up (19). Liang Y et al., in another retrospective observational study of 10 children with IgAN, reported that all patients received concomitant glucocorticoids, with some also on immunosuppressants. At a median follow-up of 3.75 months, 4 patients achieved complete remission, glucocorticoid dosage was reduced by 33.3%–92%, and 4 patients discontinued glucocorticoids entirely (20). Zhou L et al. included 13 children with advanced IgAN and IgA vasculitis nephritis (IgAVN) (7 with IgAN and 6 with IgAVN) in a single-center retrospective study, with all patients receiving telitacicept and followed for at least 6 months. Complete remission was achieved in 4 patients, with a median (IQR) reduction in urine protein-to-creatinine ratio (UPCR) of 90.74% (84.38%–94.76%), and 5 patients discontinued glucocorticoids (22). In this retrospective study of 11 children with IgAN treated with telitacicept and followed for at least 6 months, a non-significant decrease in 24-h UP was observed at week 4 (*P* > 0.05), with significant reductions at weeks 12 and 24 (*P* < 0.01) and a median reduction of 73.0% at week 24. Notably, glucocorticoid dosages and other major concomitant medications remained largely unchanged during the 1-month period prior to telitacicept treatment, whereas urinary protein levels increased significantly from 1 month before baseline to baseline (*P* = 0.037). These findings support a potential correlation between telitacicept and the observed reduction in proteinuria. Furthermore, the early downward trend at week 4 suggests a rapid therapeutic effect, consistent with observations in adult IgAN patients (18, 24–26). However, it is important to interpret these results with caution, as all pediatric studies on telitacicept in IgAN, including ours, are limited by the absence of strict controls, precluding definitive conclusions regarding its independent antiproteinuric effect.

Telitacicept may affect the levels of peripheral blood immunoglobulins in children with IgAN. The present study revealed that at the 24-week follow-up endpoint, the mean levels of IgG, IgA, and IgM were significantly lower than those at baseline, with more pronounced reductions observed for IgA and IgM. Given the mechanism of action of telitacicept (8, 27), this outcome is not difficult to understand. The degree of immunoglobulin reduction in this cohort was mild, and no severe hypogammaglobulinemia was observed during the follow-up period. No patient received exogenous intravenous immunoglobulin infusions, and no severe infections were observed. The results also revealed that the CD19+ B-cell count tended to decrease at each follow-up time point, with a significant reduction at the endpoint compared with baseline. Mechanistically, telitacicept does not directly target CD19 + or CD20 + antigen markers on the surface of mature B cells. Its effect lies in blocking BAFF, a key activating factor in the differentiation of immature B cells into mature B cells, and APRIL, a key activating factor in the differentiation of mature B cells into plasma cells (8). Theoretically, its impact on CD19 + or CD20+ B-cells is limited. However, the reduction in the total number of mature B cells may secondarily lead to a decrease in the number of CD19+ B-cells, which serve as a marker of mature B cells. This effect of reducing the number of mature B cells has also been confirmed in clinical observational studies by other investigators (28, 29).

Telitacicept appears to serve as a potential glucocorticoid-sparing agent. In the present study, a significant reduction in 24-h UP was observed as early as 4 weeks after treatment initiation, accompanied by a marked decrease in glucocorticoid dosage. During subsequent follow-up, a reduction in the use of other immunosuppressants (MMF and Tac) was also observed. Multiple pediatric studies on telitacicept in IgAN have demonstrated a reduction in glucocorticoid and immunosuppressant use (19, 20, 22, 23). However, given the short follow-up duration and the absence of a strict control group in this cohort, the inhibitory effect on immunosuppressant dosage and variety should be interpreted with caution.

The safety profile of telitacicept was also preliminarily confirmed in the present study. During the follow-up period, the eGFR remained stable in all patients, and the difference in the eGFR at the endpoint compared with baseline was not statistically significant. To a certain extent, these findings confirm the renal safety of telitacicept treatment. As a B-cell-targeting biological agent, telitacicept raises particular concerns regarding potential infectious complications, especially severe or fatal infections. According to the literature, the adverse effects of telitacicept are predominantly local skin reactions, with systemic infections being uncommon and mainly consisting of urinary tract and respiratory tract infections (17–19, 22, 23, 26). To date, no fatal infectious complications have been reported in the pediatric population. Although the present study included a small number of patients, only a few cases of upper respiratory tract infection and mild local injection site reactions were documented, and no urinary tract infection was noted, which may be attributable to adequate health education provided prior to enrollment.

## 5 Conclusions and limitations

This study provides preliminary evidence that telitacicept may be correlated with reduced proteinuria and modest decreases in immunoglobulins and CD19+ B-cell counts in pediatric IgAN. Furthermore, it may enable glucocorticoid and immunosuppressant sparing, with a favorable safety profile. However, this study has several notable limitations. First, the small sample size may introduce selection bias. Although the follow-up results suggest a significant proteinuria-reducing effect of telitacicept, this conclusion should be interpreted with caution. Second, this was a self-controlled before-and-after study lacking a strict control group. These two major limitations mean that the findings support only an association between the drug and the observed efficacy and safety outcomes but cannot be used for causal inference. Third, the observation period was relatively short, precluding validation of the medium- to long-term efficacy and safety of telitacicept. Fourth, there were multiple confounding factors beyond drug intervention, including the concomitant use of immunosuppressants such as glucocorticoids in most patients. Although these factors were partially addressed in the discussion, they may still have influenced the assessment of treatment efficacy. Therefore, the results of this study need to be further validated by rigorously designed, prospective, large-scale, controlled clinical trials.

## Data Availability

The raw data supporting the conclusions of this article will be made available by the authors, without undue reservation.
